# Pregnancy as a Period of Enhanced Risk for Non-Cavitated Caries Lesions

**DOI:** 10.3290/j.ohpd.a44445

**Published:** 2020-07-04

**Authors:** Rute Rio, Benedita Sampaio-Maia, Maria Lurdes Pereira, Mário Jorge Silva, Álvaro Azevedo

**Affiliations:** a Dentist, Faculdade de Medicina Dentária, Universidade do Porto, Porto, Portugal; Universidade Católica Portuguesa – Campus Viseu, Portugal. Study conception and design; data acquisition, analysis and interpretation, and writing, editing, reviewing and final approval of article.; b Microbiologist, Faculdade de Medicina Dentária, Universidade do Porto, Porto, Portugal; INEB-Instituto de Engenharia Biomédica, Universidade do Porto, Portugal. Instituto de Investigação e Inovação em Saúde, Universidade do Porto, Portugal; study conception and design; data analysis and interpretation, and writing, editing, reviewing and final approval of article.; c Assistant Professor, Faculdade de Medicina Dentária, Universidade do Porto, Porto, Portugal; EPIUnit – Institute of Public Health, Universidade do Porto, Portugal. Data analysis and interpretation, and writing of the article; contributed to the discussion and final approval of the article.; d Full Professor, Faculdade de Medicina Dentária, Universidade do Porto, Porto, Portugal; EPIUnit – Institute of Public Health, Universidade do Porto, Portugal. study conception and design; editing, reviewing and final approval of article.; e Assistant Professor, Faculdade de Medicina Dentária, Universidade do Porto, Porto, Portugal; EPIUnit – Institute of Public Health, Universidade do Porto, Portugal. Study conception and design; data analysis and interpretation, and writing, editing, reviewing and final approval of article.

**Keywords:** pregnancy, dental caries, saliva pH, oral hygiene, diet

## Abstract

**Purpose:**

To investigate if pregnancy represents a period of increased risk of non-cavitated dental caries related to changes in saliva and oral health behaviours.

**Materials and Methods:**

A non-randomised longitudinal study was performed with 27 pregnant women and 25 non-pregnant women, who were evaluated twice with the same time gap (24 weeks on average). At the first visit sociodemographic and oral health-related behaviours were assessed through a structured questionnaire. At the second visit changes related to eating sweet snacks and oral hygiene habits were also assessed. In both visits the surface-related caries status was evaluated according to ICDAS II criteria. Calculation of D_0_ (Sound), D_1–2_ (visual changes) and D_3–4_ (precavitated caries lesions) Index was based on data collected from clinical examination. Saliva pH and saliva flow rate were also assessed.

**Results:**

Throughout pregnancy, a statistically significant increase of eating sweet snacks between main meals was reported, with no effective adaptation of oral hygiene habits. In comparison to the non-pregnant group, pregnant women presented a lower saliva pH at both the first and second visit, p < 0.0005. During the follow-up period, a decrease in the frequency of caries-free surfaces was observed in the pregnant women (p = 0.004) and an increase in precavitated caries lesions (p = 0.011).

**Conclusion:**

The main results support the hypothesis that during pregnancy women are prone to enamel demineralisation, namely, to exhibiting additional lesions characterised by precavitated caries lesions.

Remarkable physiological and hormonal adjustments occur during pregnancy. Although other hormonal changes also take place, the most statistically significant is the increased production of oestrogens and progesterone.^12,21,39^ Besides the modulation of the immune system during pregnancy, these two hormones have effects on the vascular system, inducing an increase in gingival inflammation, in gingival bleeding and in crevicular fluid flow.^12,24^ The oral pathologies frequently described in pregnant women are associated with periodontal health, and include pyogenic granuloma, periodontitis and gingivitis.^12,14,21,28,30,32,39^

In addition, as the basal metabolic rate rises, more energy is needed for the growing foetus, the sweet taste preference becomes more frequent in the early pregnancy.^9,41^ Consequently, a dietary re-adaptation of the pregnant women is commonly observed, such as an increase in the consumption of carbohydrates, sugary snacks and drinks.^14,21,27,39^ However, regular toothbrushing may be significantly compromised during pregnancy mainly due to nausea and sickness, which are common symptoms in early pregnancy.^12,30^ Furthermore, during pregnancy there is frequently a reduction in dentist appointments for treatments or check-up due to beliefs, fear or myths about the safety of dental care during this period, which might increase the risk of dental caries.^18,33,38,42^

The oral environment is also subject to changes during pregnancy. Although reports on sialometric and sialochemical alterations during pregnancy are not consensual, some changes may promote enamel demineralisation and impairment of its remineralisation, such as acidic saliva pH, the increment of plaque acidogenicity, decreased buffer effect and altered levels of calcium and phosphate in whole saliva.^14,19–21,34,41^ Moreover, a decrease in saliva flow rate is described, which may lead to an increase in the retention of carbohydrates on tooth surfaces.^22^

Hormonal changes, together with the oral biochemical alterations, frequent gastric acid reflux, and inadequate attention to oral health, may contribute to an oral microbiota modification.^39^ Indeed, overgrowing *Lactobacillus* and *Streptococcus mutans* capable of metabolising oestradiol have been detected during pregnancy and during lactation.^21,30^

Factors such as the use of fluoride in toothpaste, oral hygiene habits, dietary habits, smoking habits, level of education and access to dental care are relevant variables when assessing the risk of dental caries. Moreover, although the reduction of oral pH is correlated to the number of initial caries lesions^2^ and the most important risk factors for dental caries may be present during pregnancy, it has not been properly established that pregnant women are more susceptible to developing caries.^7,12,14,19–21,24,28, 30,32,34,39^ Thus, the aim of this study was to investigate if pregnancy represents a period of increased risk of non-cavitated dental caries related to changes in saliva and oral health behaviours.

## Materials and Methods

**Fig 1:**
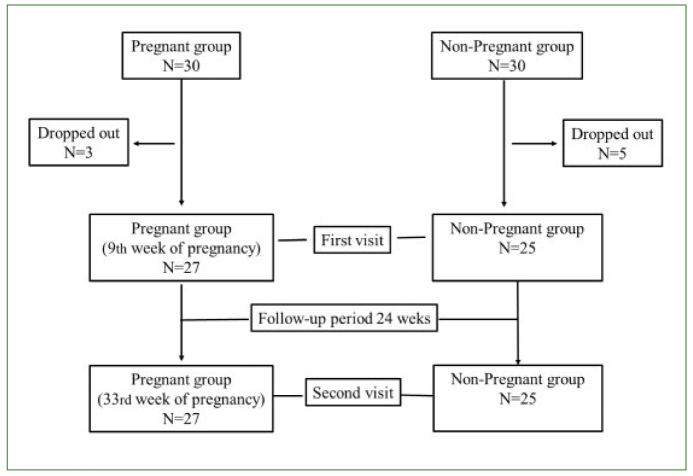
Study flow diagram.

A longitudinal study was performed with a non-randomised sample divided into two groups (Fig 1). One group was composed of 30 pregnant women aged between 23 and 40 years, who attended the outpatient clinic of the Department of Obstetrics and Gynaecology of Arrábida Hospital, Porto, Portugal, around the ninth week of pregnancy (first visit) and thirty-third week of pregnancy (second visit) for routine obstetric examination (24 week gap on average – follow-up period) during 2015. The control group comprised 30 non-pregnant women aged between 24 and 39 years old attending the same department for routine gynaecological examination, who were evaluated twice in the same time gap as pregnant women, also in 2015. Due to a change of hospital, three pregnant women and five controls dropped out of the study. Exclusion criteria included high-risk pregnancy, patients with less than 16 teeth, menopausal subjects, drug addiction and subjects presenting compromising systemic diseases. For both groups, during the first visit a structured questionnaire was applied relating to sociodemographic questions, such as age and educational level, as well as health history. In addition, oral health behaviours such as smoking habits, daily toothbrushing, fluoride toothpaste use, the habit of eating sweet snacks and attendance for dental check-up or treatments in the previous 6 months were evaluated. At the second visit, increased frequency of eating of sweet snacks, toothbrushing habits and access to dental care during the follow-up period were assessed in the pregnant participants.

Dental caries was identified by clinical examination during the first and second visit. Caries registration was carried out according to ICDAS II criteria^13^ by an experienced examiner, previously calibrated by an expert. For interexaminer accuracy and intraexaminer reliability, a weighted Kappa value of 0.85 and 0.89 was found, respectively. Professional oral cleaning was performed before the clinical examinations. After that, each surface was examined after drying and wetting accordingly to ICDAS II criteria. Rough surfaces and pit areas were detected by gentle passing of a CPI probe. Each tooth surface was classified according to the ICDAS II scale: D_0_ – Sound (caries-free) surface, D_1_ – First visual change in enamel, D_2_ – Distinct visual change in enamel, D_3_ – Localised enamel breakdown due to caries with no visible dentine or underlying shadow, D_4_ – Underlying dark shadow from dentine with or without localised enamel breakdown. Calculation of D_0_ (sound surface), D_1–2_ (visual changes) and D_3–4_ (precavitated lesions) were based on data collected from clinical examination. In order to evaluate the progress of the caries for both groups, the difference of caries index between the first and the second visit was calculated.

Saliva was collected at both visits before the intraoral exam in a quiet room between 8:00 and 12:00 a.m. to minimise the effects of the circadian rhythm, and at least 2 h after eating, brushing teeth or the use of a mouth wash. To collect unstimulated whole saliva, the participants were asked to spit into a sterile plastic container as the saliva had accumulated in the floor of the mouth over a 5-min period. The total amount of saliva collected was registered, and the salivary flow rate (ml/min) was calculated.^29^ The salivary pH was measured immediately after saliva collection using pH indicator paper (5.0–8.0, Duotest, Germany).^3^

The research protocol was in compliance with the World Medical Association Declaration of Helsinki (version 2008) and was approved by the Ethics Committees of the Faculty of Dental Medicine and Arrábida Hospital. After the process had been explained, written informed consent was obtained from all individual participants included in the study. Confidentiality of all information was guaranteed at storage and processing stages.

The statistical analysis was performed using the IBM SPSS Statistics for Windows (V.25.0), the chi-square test, the Mann–Whitney U test, and the Wilcoxon test for paired samples, and the binomial exact test was applied in order to compare the observed frequencies of the two categories of dichotomous variables with the frequencies that were expected. A statistical significance level of 0.05 was considered.

## Results

Table 1 shows the results regarding sociodemographic and oral health-related habits at the first evaluation. Both pregnant and non-pregnant groups were similar regarding the sociodemographic characteristics such as age (p = 0.748) and university studies (p = 0.282). Behavioural determinants of caries development were also assessed. The percentage of smokers, the frequency of toothbrushing, the use of fluoride toothpaste and snack consumption did not reveal statistically significant differences between pregnant and non-pregnant women.

**Table 1:** Sociodemographic factors and oral health-related behaviours in pregnant and non-pregnant women at the first visit

	Non-pregnant (N = 25)	Pregnant (N = 27)	P values
**Age (years)**	32.6 ± 4.5	32.3 ± 4.1	0.748^a^
**University degree**			
Yes	14 (56.0%)	19 (70.4%)	0.282^b^
No	11 (44.0%)	8 (29.6%)	
**Smokers**			
Yes	4 (16.7%)	1 (3.7%)	0.175^b^
No	20 (83.3%)	26 (96.3%)	
**Toothbrushing per day**			
Twice or more	24 (96.0%)	21 (77.8%)	0.129^b^
Less than twice	1 (4%)	6 (22.2%)	
**Fluoride toothpaste**			
Yes	23 (92.0%)	20 (74.1%)	0.180^b^
No	2 (8%)	7 (25.9%)	
**Sweet snacking**			
Yes	16 (64.0%)	20 (74.1%)	0.432^b^
No	9 (36.0%)	7 (25.9%)	

Results expressed in prevalence: N (%) or as mean ± SD. P values were calculated using ^a^ Mann–Whitney U test and ^b^ chi-square test.

The majority (88.9%) of the pregnant women declared that they did not have any dental check-up in the previous 6 months and 96.3% had not undergone any dental treatment. Between the first and second visit, no dental fillings or dental extractions were performed in either group.

Changes in oral health behaviours during the follow-up period were also assessed and registered (Table 2). At the second visit, all non-pregnant women reported that they had not changed their oral health behaviours during the follow-up period. A minority of pregnant women (26%) reported that they had increased their oral hygiene habits during pregnancy (p < 0.0005). However, in the same time gap, the majority of pregnant women (63%) had increased their habit of eating sweet snacks (p = 0.005).

**Table 2:** Increase in frequency of toothbrushing and sweet snacks consumption in pregnant women during the follow-up period

	Toothbrushing	Sweet snacks consumption
Increasing	26.0%	63.0%
p values	< 0.0005	0.005

results expressed in prevalence (%); p values were calculated using Binomial exact test (one tailed)

Regarding non-stimulated saliva flow rate, no statistically significant differences were observed between pregnant and non-pregnant women throughout the study (Table 3). In comparison to the non-pregnant group, pregnant women presented a lower saliva pH at both the first and second visit, p < 0.0005. No statistically significant differences were observed in saliva pH between the first and second visit for either group (Table 3).

**Table 3:** Comparisons of saliva flow rate and pH between pregnant and non-pregnant group at first visit and second visit and within groups between the first visit and second visit

	1^st^ visit	2^nd^ visit	p values^a^
**Saliva flow rate (ml/min)**			
Non-pregnant	1.71 ± 0.46	1.78 ± 0.48	0.318
Pregnant	1.86 ± 0.49	1.89 ± 0.40	0.776
p values^b^	0.304	0.457	
**Saliva pH**			
Non-pregnant	7.04 ± 0.28	7.03 ± 0.24	0.782
Pregnant	6.69 ± 0.35	6.73 ± 0.28	0.433
p values^b^	< 0.0005	< 0.0005	

Results expressed in mean ± SD. ^a^ Differences between first and second visit, Wilcoxon signed rank test. ^b^ Differences between non-pregnant and pregnant women, Mann–Whitney U test.

During the follow-up period, a decrease in the frequency of caries-free surfaces in both groups was seen (Table 4), attaining statistical significance only in the pregnant women (p = 0.004). For the same time gap, the non-pregnant group registered an increment for D_1–2_ and a reduction for D_3–4_ with no statistical significance. On the other hand, the pregnant group achieved a statistically significant decrease for D_1–2_ (p = 0.021) and an increment for D_3–4_ (p = 0.011) (Table 4).

**Table 4:** Comparisons of caries-free (D_0_), visual changes (D_1–2_) and precavitated lesions (D_3–4_) according to ICDAS II criteria between the first visit and second visit in non-pregnant and pregnant groups

	1^st^ visit	2^nd^ visit	p values^a^
**Caries-free **(D_0_)			
Non-pregnant	123.36 ± 8.48	123.28 ± 8.48	0.250
Pregnant	125.22 ± 10.07	124.93 ± 10.09	0.004
**Visual changes **(D_1–2_)			
Non-pregnant	1.24 ± 1.39	1.32 ± 1.44	0.500
Pregnant	2.26 ± 1.95	1.78 ± 1.85	0.021
**Precavitated lesions **(D_3–4_)			
Non-pregnant	2.28 ± 1.62	1.92 ± 1.55	0.063
Pregnant	1.37 ± 1.84	1.93 ± 1.62	0.011

Results expressed in mean ± SD. ^a^ Differences between first and second visit, Wilcoxon signed rank test.

Table 5 illustrates the differences (1^st^–2^nd^ visit) related to D_1–2_ and D_3–4_ for both groups of women. The non-pregnant group achieved a negative difference between the first and second visit, namely an increment of D_1–2_ in the follow-up period (–0.08 ± 0.28). Conversely, the pregnant women registered a decrease (0.48 ± 0.98) in the same time gap, and the difference was statistically significant (p = 0.012). Regarding the difference of D_3–4_ between the first and second visit, for the groups in comparison, the opposite was observed and it was statistically significant (p = 0.001).

**Table 5:** Comparison of differences of D_1–2_ surfaces and D_3–4_ surfaces observed during the first visit and second visit between non-pregnant and pregnant group

	Non-pregnant 1^st^–2^nd^ visit	Pregnant 1^st^–2^nd^ visit	p values^a^
D_1–2_	–0.08 ± 0.28	0.48 ± 0.98	0.012
D_3–4_	0.36 ± 0.81	–0.56 ± 1.01	0.001

Results expressed in mean ± SD. P values were calculated using ^a^ Mann–Whitney U test.

## Discussion

The results support the hypothesis that, during the follow-up period, pregnant women were more prone to enamel demineralisation than non-pregnant women, namely to exhibiting additional lesions D_3–4_ characterised by precavitated caries lesions. In addition, pregnant women presented a greater reduction of D_0_ than the control group, during the same time period.

Dental caries result from the demineralisation of tooth tissues.^8,10,31^ The demineralisation and remineralisation unbalance is, in large measure, attributed to the acids produced from the microbial fermentation of dietary carbohydrates in biofilm, inducing a pH decrease.^2,11,16,41,45^ In this way, a lower saliva pH in non-stimulated saliva in pregnant women in comparison to the control group, as observed in the present study, may explain the greater incidence of D_3–4_ caries lesions obtained in the group of pregnant women during the follow-up period. Aranibar Quiroz (2014) showed that a strong decrease in pH plaque was observed when adolescents were submitted to a sucrose rinse. Moreover, in the same study a strong correlation was observed with initial caries and not with decayed teeth presenting manifest caries. However, a pH reduction was not found in the pregnant participants between the first and the second visit, unlike other authors who found a reduction in the third trimester of pregnancy and after childbirth.^16^ Still, it has been stated that the decrease of pH may occur in the early periods of pregnancy when feeding and hygiene habits change and proliferation of oral microorganisms increases.^11,21,39^ Biochemical saliva changes have been reported throughout pregnancy, namely in calcium/phosphate levels^34^ and in non-stimulated saliva flow rate,^19^ although no statistical difference in non-stimulated saliva flow rate was observed between pregnant and non-pregnant women in the present study.

As has been stated by other authors,^14,21,39^ the majority of pregnant women alter their food intake, consuming more sweet snacks between main meals throughout their pregnancy. However, during the same period no improvement in oral hygiene habits is observed for the majority of women. In addition to other factors, the lack of adaptation of oral hygiene strategies by pregnant women can be explained, by the dislike of the taste of toothpaste, nausea and sickness.^12,30^ Indeed, changes in smell and taste perception occur frequently during the early weeks of the pregnancy.^6,25,40^

Moreover, during pregnancy, hormone-related changes occur and a higher susceptibility to periodontal diseases and consequently gingival bleeding is currently widely accepted.^14,21,28,30,39^ Such circumstances are compounded by the fear of brushing in that situation, lack of knowledge regarding the safety of treatment or fear of malpractice.^4,33,35,38,42,43^ Accordingly, the results showed that all undergo any check-up or dental care in the previous months.

Differences in methodological procedures may explain why the results regarding the relationship between pregnancy and dental caries are not consistent with those obtained by other authors.^4,26,28,32^ On the one hand, such disagreement may occur due the less sensitive methods used to assess dental caries.^26,28^ Indeed, there is scientific evidence that at least half of the total number of caries experienced will remain undetected if the criteria include only distinct cavities, which would be insufficient to assess the evolution of dental caries, which in general have a slower rate of progression.^1,23^ Notwithstanding, the clinical examination using the World Health Organization (WHO) diagnostic criteria, applied by other authors, did not prevent them from obtaining an increase of prevalence of caries in pregnant women in comparison to non-pregnant women.^26,32^ Such results can be explained by the representativeness of the sample, since in these cross-sectional studies, pregnant women had a low educational level, poor hygiene habits as well as an inadequate practice of dental healthcare.^26,32^

It is known that for epidemiological risk assessment, longitudinal studies, such as the one we adopted, are preferable to cross-sectional studies. So, in comparison to previous reports, this prospective approach using a sensitive caries evaluation methodology (ICDAS) and the characterisation of saliva biochemistry, as well as the associated behavioural risk factors allow us to improve knowledge on caries onset and progression throughout pregnancy. Nevertheless, in the present study, the internal validity could be compromised due the sample size and the non-randomised sampling technique used, which increases the beta error and calls into question the representativeness of the sample, respectively. Nonetheless, at the first visit, the comparability of the two groups under study is not compromised in this way, since the sociodemographic and oral health behaviour confounders such as educational level, smoking habits, frequency of brushing teeth, fluoride exposure and eating sweet snacks did not attain statistically significant differences. For future studies, it would be interesting to improve the power of the tests. Additionally, it would be interesting to include the characterisation of the bacterial profile to evaluate the acidogenicity of the plaque and the incidence of caries during pregnancy and after delivery.

Despite the biological explanations, the higher caries risk for pregnant women can be explained by changes in dietary habits with no effective adaptation of oral hygiene strategies. Furthermore, findings relating the increased parity with untreated decay and tooth loss, that can lead to a higher treatment needs after delivery, reinforce that greater caries susceptibility cannot be exclusively explained by biological factors.^4,5,7,17,36,37^ Regular access to dental care, oral hygiene education, motivation and awareness-raising among pregnant women are recommended even after delivery, in order to improve oral health-related quality of life, but also to reduce potential complications during pregnancy and the risk of the child developing early childhood caries.^15–17,19,28,32,41,44^

The present results support the hypothesis that, due to biological and behavioural factors, and without implementation of additional preventive measures, the period of pregnancy is prone to enamel demineralisation, namely to the development of additional lesions D_3–4_ characterised by precavitated caries lesions.
